# Fully immersive virtual reality exergames with dual-task components for patients with Parkinson’s disease: a feasibility study

**DOI:** 10.1186/s12984-023-01215-7

**Published:** 2023-07-18

**Authors:** Seo Jung Yun, Sung Eun Hyun, Byung-Mo Oh, Han Gil Seo

**Affiliations:** 1 Department of Rehabilitation Medicine, Seoul National University College of Medicine, Seoul National University Hospital, Seoul, Republic of Korea; 2 Department of Human Systems Medicine, Seoul National University College of Medicine, Seoul, Republic of Korea; 3 National Traffic Injury Rehabilitation Hospital, Yangpyeong, Republic of Korea

**Keywords:** Dual-task training, Exergaming, Parkinson’s disease, Virtual reality

## Abstract

**Background:**

Dual-task training in Parkinson’s disease (PD) improves spatiotemporal gait parameters, cognition, and quality of life. Virtual reality (VR) has been used as a therapeutic tool for patients to participate in activities in a safe environment, engage in multisensory experiences, and improve motivation and interest in rehabilitation. This study aimed to investigate the feasibility of fully immersive VR exergames with dual-task components in patients with PD.

**Methods:**

We developed VR exergames (go/no-go punch game, go/no-go stepping game, and number punch game) to improve habitual behavior control using motor–cognitive dual-task performance in patients with PD. The participants underwent 10 sessions 2–3 times a week, consisting of 30 min per session. The Unified Parkinson’s Disease Rating Scale, Timed Up and Go test (TUG) under single- and dual-task (cognitive and physical) conditions, Berg balance scale (BBS), Stroop test, trail-making test, and digit span were evaluated before and after intervention. The Simulator Sickness Questionnaire (SSQ) was used to assess VR cybersickness. Usability was assessed using a self-reported questionnaire.

**Results:**

Twelve patients were enrolled and completed the entire training session. The mean age of participants was 73.83 ± 6.09 years; mean disease duration was 128.83 ± 76.96 months. The Hoehn and Yahr stages were 2.5 in seven patients and 3 in five patients. A significant improvement was observed in BBS and Stroop color–word test (*p* = 0.047 and *p* = 0.003, respectively). TUG time and dual-task interferences showed positive changes, but these changes were not statistically significant. The median SSQ total score was 28.05 (IQR: 29.92), 13.09 (IQR: 11.22), and 35.53 (IQR: 52.36) before, after the first session, and after the final session, respectively; the differences were not significant. Overall satisfaction with the intervention was 6.0 (IQR: 1.25) on a 7-point Likert-type scale.

**Conclusions:**

Fully immersive VR exergames combined with physical and cognitive tasks may be used for rehabilitation of patients with PD without causing serious adverse effects. Furthermore, the exergames using dual-task components improved executive function and balance. Further development of VR training content may be needed to improve motor and dual-task performances.

*Trial registration* NCT04787549 (https://clinicaltrials.gov/ct2/show/NCT04787549)

**Supplementary Information:**

The online version contains supplementary material available at 10.1186/s12984-023-01215-7.

## Background

Parkinson’s disease (PD) is a progressive neurodegenerative disease characterized by difficulties in initiating, executing, and inhibiting voluntary movements [1, 2]. In the initial stages of PD, sensorimotor circuits of the basal ganglia are affected due to the loss of dopaminergic neurons in the ventrolateral substantia nigra and their terminals in the caudolateral sensorimotor putamen [3]. Thus, patients with PD rely on goal-directed control, which is associated with basal ganglia networks, instead of the negatively affected habitual control pathway [4]. The goal-directed mode, slow and serial, requires immense cognitive effort compared to stimulus response-habitual responding, making the behavior of patients with PD more susceptible to interference from other goal-oriented tasks [5]. Consequently, the deterioration in movement automaticity decreases dual-task performance [6].

Performing two or more tasks simultaneously is required in daily life and allows people to perform activities, such as walking and talking [7]. Because walking relies on high-level functioning of the neurological system and cognitive process, gait disturbance in PD may be aggravated when walking is combined with a concurrent goal-directed task [8]. Dysfunction in dual-task performance in PD is associated with a higher fall risk, reduced functional activities of daily living, and lower quality of life [9, 10]. Several therapeutic methods have been attempted to remedy the impaired dual-task performance.

Physical training combined with cognitive tasks has shown positive effects on spatiotemporal gait parameters under dual-task conditions [11]. Likewise, several studies have demonstrated that dual-task training in PD led to greater improvement in gait velocity, step length, cognition, and dual-task gait speed without increasing the risk of falls [12, 13]. Additionally, a cognitively challenging exercise program that targets both executive function/attention and physical function improved balance, cognitive-gait interference, independence, and quality of life [14]. Despite the advantages of dual-task training, developing and implementing a well-organized training program in clinical environments is challenging because of the absence of a gold standard, difficulties in setting up programs, and dependence on therapist capacity. Therefore, there might be unmet needs for specialized dual-task intervention programs for patients with PD.

Virtual reality (VR) is a technology that creates a computer-generated artificial environment that can be configured easily for therapeutic purposes [15]. VR has been used as a promising rehabilitation therapeutic tool for patients to provide a safe environment to participate in activities, offer multisensory experiences, and induce interest in patients with neurological diseases [16]. According to the immersion level, VR can be classified as non-immersive, semi-immersive, or fully immersive. A higher immersion level allows patients to readily focus on therapy without external interference [17]. VR exergames, which integrate gaming with physical exercise, may offer a more engaging and interactive approach to physical fitness [18]. Studies have shown that VR exergames can potentially increase energy expenditure and improve various physical fitness measures [19]. The VR environment allows for a wide range of virtual activities, including sports, dance, and adventure games [20]. This variety helps to overcome boredom and to maintain motivation, leading to increased adherence to physical activity. Furthermore, applying VR technology also has the advantage of relieving the burden on healthcare professionals and increasing their efficacy [21].

Recent meta-analyses have demonstrated that VR for PD positively affects gait function, balance, and quality of life similar to conventional physiotherapy [22, 23]. However, most studies used non-customized commercial game platforms, such as Nintendo Wii™, non-immersive or semi-immersive VR hardware, and measurements that focused only on motor function. Therefore, we developed customized VR exergames that integrate cognitive and motor tasks to improve physical and cognitive function along with dual-task performance and investigated the feasibility of fully immersive VR exergames for patients with PD.

## Methods

### Study design and participants

This was a prospective, single-center, single-arm feasibility study. The study protocol was approved by the Institutional Review Board of the Seoul National University Hospital (IRB No. 2010-132-1167) and registered at ClinicalTrials.gov (NCT 04787549). The study was performed in accordance with the principles of Good Clinical Practice and the Declaration of Helsinki.

The inclusion criteria were patients (1) aged > 18 years; (2) clinically diagnosed with idiopathic PD; and (3) modified Hoehn and Yahr (H&Y) stages 2, 2.5, or 3. Exclusion criteria were (1) moderate to severe cognitive impairment based on the mini-mental state examination (MMSE) score (< 20); (2) severe dyskinesia or “on–off” fluctuations; (3) plan to adjust PD medication during patient screening; (4) other brain diseases, including stroke and brain tumors; (5) seizure history; (6) vestibular disorders or paroxysmal vertigo; and (7) other comorbidities that may limit participation in the study.

The study participants were recruited through the outpatient clinic of Seoul National University Hospital. All the participants provided written informed consent. Every evaluation and intervention proceeded at the “on” state, which is the peak effect of PD medication. Antiparkinsonian medications were maintained at the same doses during the study period for all participants.

### Exergames developed in the study

We developed fully immersive VR exergames with dual-task components for patients with PD. Three exergames (go/no-go punch game, go/no-go stepping game, and number punch game) aimed to improve habitual behavior control using gross motor activities combined with the cognitive task in PD. Physical exercise in patients with PD is emphasized as a means of maintaining physical function and inducing neuroplasticity in motor and cognitive neural networks [24]. Recently, boxing has gained worldwide recognition as a potential therapeutic intervention for PD. Boxing involves movement of all parts of the body in a weight-bearing and aerobic manner [25]. The leg agility assessment, which is part of the Unified Parkinson’s Disease Rating Scale (UPDRS), ascertains the speed, consistency, and range of motion of each leg individually by requiring the patient to lift and stomp their feet on the ground [26]. For this reason, we chose boxing and leg agility movements for upper and lower extremity activities. The go/no-go paradigm stimulates inhibition response and cognitive flexibility in patients with PD by requiring instantaneous decision-making [27]. Several studies have also demonstrated the positive effects of boxing interventions that include cognitive challenges (go/no-go task) for patients with PD [14, 28]. However, implementing these programs in clinical settings can be challenging because they require large spaces, numerous supplies, and place a significant burden on healthcare professionals. To overcome these limitations, we combined gross motor activities and cognitive tasks with VR which could enhance motivation and interest, and surpass space restrictions. The level of difficulty (stages) was established based on the number and complexity of conditions. The exergames have been developed for gameplay while seated. Seated VR exergames could reduce the risk of falls, prevent players from injuries by bumping into nearby objects, and allow physically challenged users (such as the elderly or wheelchair users) to participate in the game safely [29].

The VR environment consisted of a virtual gym with a treadmill, a yoga mat, steppers, and dumbbells. When the game started, instructions per stage were provided verbally and as text on a green board in front of the patients. Each trial could be set 10–50 times by a therapist. Patients were required to respond within 3 s after a command; success or failure to follow the command was then recorded. Background music, of which there are several, was used as an obstructive factor when patients got used to games. The number of correct and incorrect responses, response times, play modes, play stages, and total playtime were recorded.

**Fig. 1:**
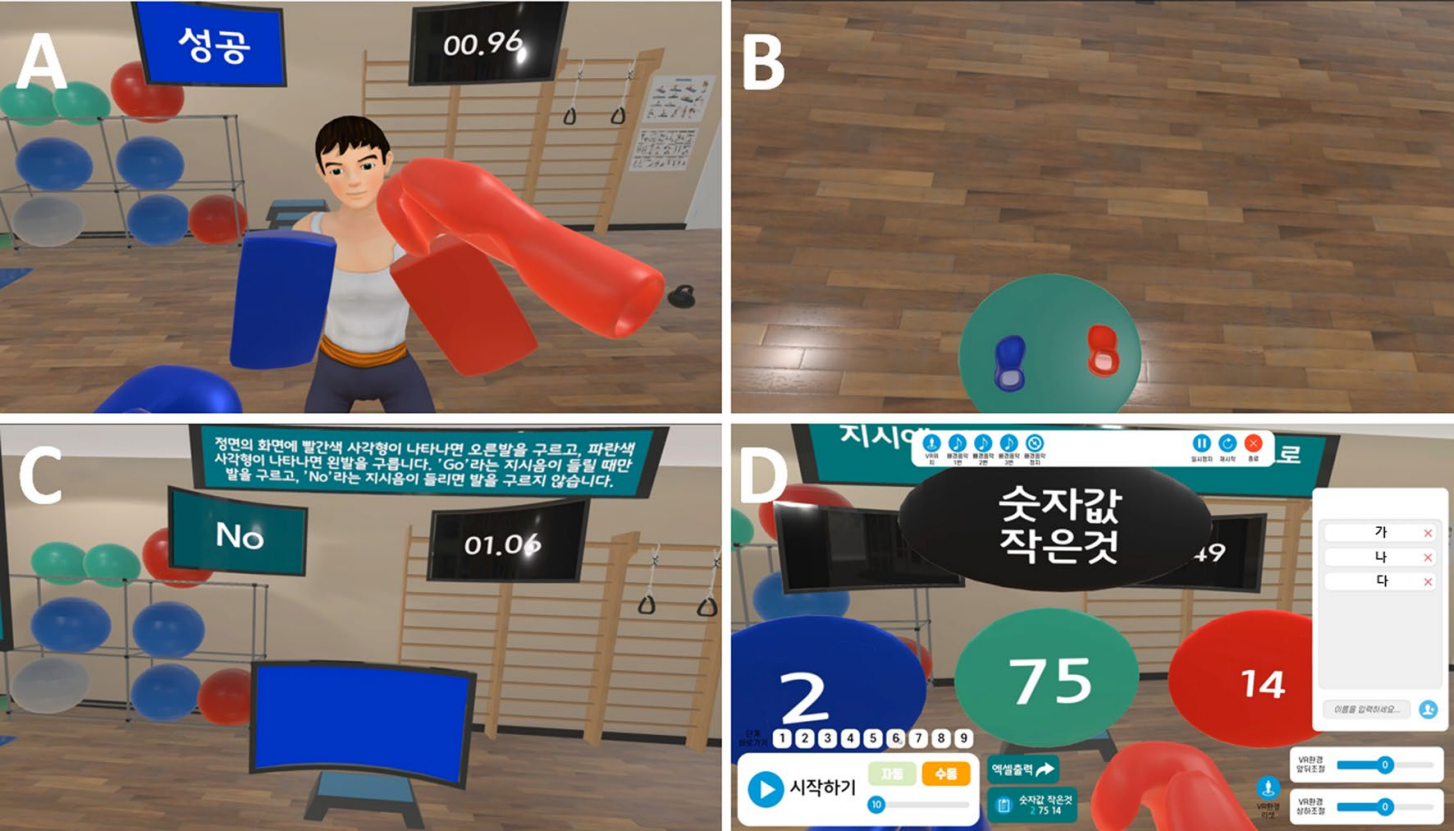
Virtual reality exergames. **A** The go/no-go punch game. Instructions are on the left monitor, and time elapsed is on the right monitor. **B, C** The go/no-go stepping game. **D** Therapist’s view of the number game. Stages, number of games, position of patients/objects, and background music can be adjusted. Also, a therapist can confirm the correct answers

The go/no-go punch game consisted of nine stages (Table 1). In the VR environment, a coach wearing boxing pads on both hands stood in the middle of the gym (Fig. 1A). The coach stretched his left or right arm toward the patient during the game. Depending on the instructions in each stage, the patient punched or did not punch the pads. The ratio of “go” or “no-go” was set to 50:50. The primary color of the pads in stages 1–3 was blue and red, while in stages 4–9, the pads’ default color was grey, which randomly changed to red or blue when the coach stretched his arm out. The controllers vibrated and provided sensory feedback when patients hit the pads.

**Table 1:** Instructions of the virtual reality exergames developed in the study

Stage	Instruction
Go/no-go punch game	
1	Punch the boxing pad with your left and right fists alternately
2	Punch the boxing pad when you hear “go”; do not hit it when you hear “no.”
3	Punch the boxing pad when you hear “no”; do not hit it when you hear “go.”
4	Punch the red boxing pad using the right fist and strike the blue pad using the left fist
5	Punch the blue boxing pad using the right fist and strike the red pad using the left fist
6	Punch the red boxing pad using the right fist and strike the blue pad using the left fist. Hit the boxing pad when you hear “go”; do not hit it when you hear “no.”
7	Punch the red boxing pad using the right fist and strike the blue pad using the left fist. Hit the boxing pad when you hear “no”; do not hit it when you hear “go.”
8	Punch the blue boxing pad using the right fist and strike the red pad using the left fist. Hit the boxing pad when you hear “go”; do not hit it when you hear “no.”
9	Punch the blue boxing pad using the right fist and strike the red pad using the left fist. Hit the boxing pad when you hear “no”; do not hit it when you hear “go.”
Go/no-go stepping game	
1	Alternately stomp your left and right feet as hard as possible
2	Stomp your left and right feet as hard as possible when you hear “go”; and do not move when you hear “no.”
3	Stomp the feet as hard as possible when you hear “no”; and do not move when you hear “go.”
4	Stomp your right foot when you see a red screen and your left foot when you see a blue screen
5	Stomp your left foot when you see a red screen and your right foot when you see a blue screen
6	Stomp your foot when you see a red screen and stomp your left foot when you see a blue screen. Stomp your foot when you hear “go”; and do not move when you hear “no.”
7	Stomp your foot when you see a red screen and stomp your left foot when you see a blue screen. Stomp your foot when you hear “no”; and do not move when you hear “go.”
8	Stomp your foot when you see a blue screen and stomp your left foot when you see a red screen. Stomp your foot when you hear “go”; and do not move when you hear “no.”
9	Stomp your foot when you see a blue screen and stomp your left foot when you see a red screen. Stomp your foot when you hear “no”; and do not move when you hear “go.”
Number punch game	
1	Punch the circle with the bigger or less number as instructed (two targets)
2	Punch the circle with the biggest or least number as instructed (three targets)
3	Punch the circle with the larger or smaller font size as instructed (two targets)
4	Punch the circle with the largest or smallest font size as instructed (three targets)
5	Punch the circle with the bigger or less number, or the larger or smaller font size as instructed (two targets)
6	Punch the circle with the biggest or least number, or the largest or smallest font size as instructed (three targets)

The go/no-go stepping game also consisted of nine stages (Table 1). During the game, the patient wore trackers on both sides of the ankle. When the patient wearing a head-mounted display (HMD) looked down at his feet, he could see two shoes (Fig. 1B). A television screen was in front of the patient (Fig. 1C). The television screen randomly showed a blue or red screen from stages 4 to 9. The rules of the go/no-go stepping game were similar to those of the go/no-go punch game.

The number punch game consists of six stages (Table 1). The patient played a number punch game using two controllers. During the game, three or four circles were observed by the patient (Fig. 1D). The black topmost circle indicated the commands that the patient should execute. The commands required discrimination of quantity or font size of the number shown. According to the command, the patient had to punch a blue, green, or red circle. In the blue, green, and red circles, random numbers ranged from 1 to 99. As with the go/no-go punch game, the controllers vibrated to provide sensory feedback to the patients when they hit a circle.

### Intervention

**Fig. 2:**
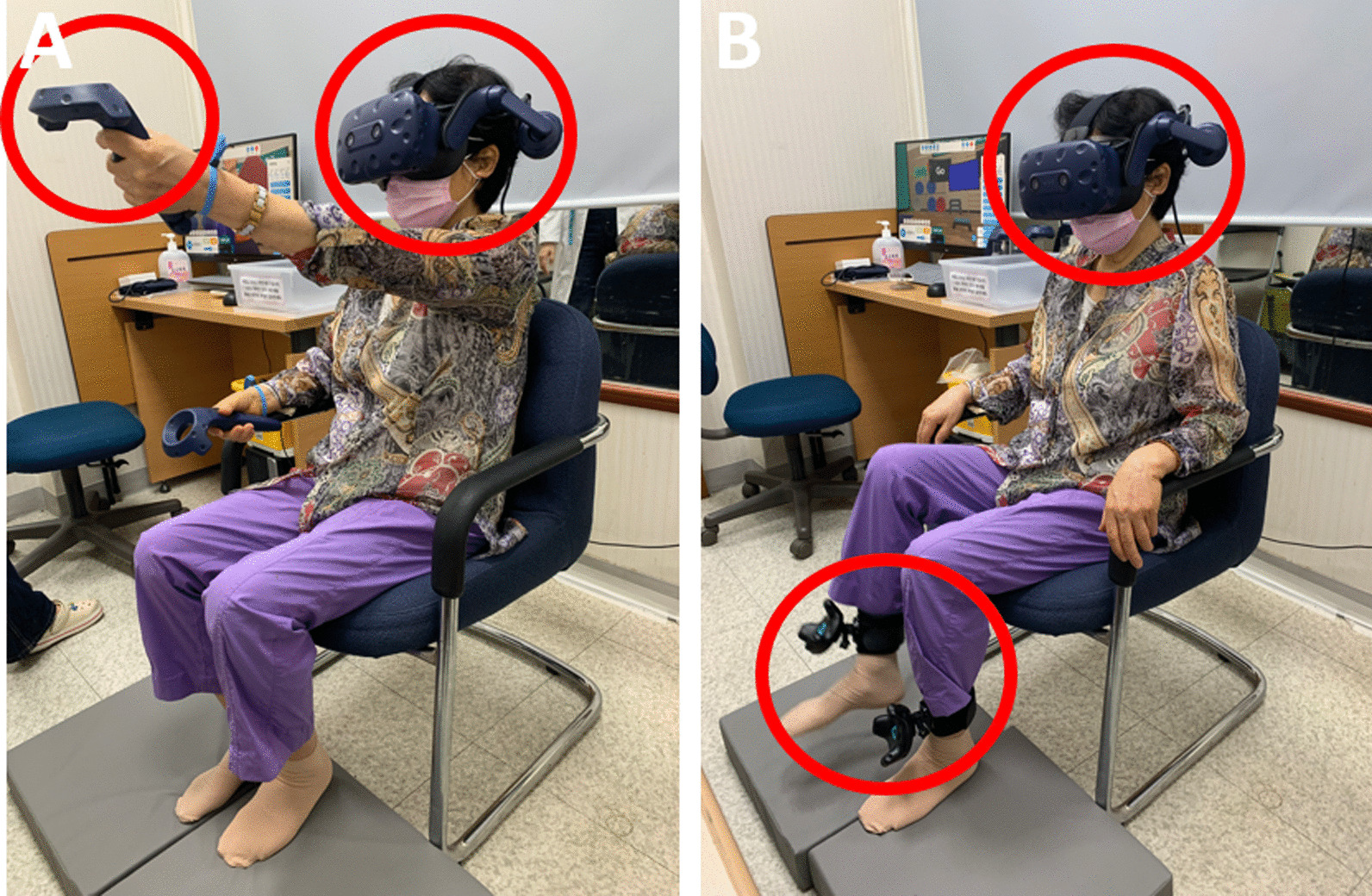
The participants wearing the head-mounted display and performing the exergames. **A** The go/no-go punch game with two controllers. **B** The go/no-go stepping game with two trackers

The HTC Vive Pro (HTC Corporation, Xindian City, Taipei), which included an HMD, controllers, trackers, and Steam VR base station 1.0, was used as the interface system. In every session, we provided disposable HMD masks to the participants and sanitized controllers and trackers after the sessions. The intervention was conducted in a sitting position while wearing the HMD and was supervised by an occupational therapist (Fig. 2). The intervention was executed on the premise that the therapist would progress to the subsequent stage if the patient demonstrated a successful completion rate of 80% or more in the preceding stage. Once the participants became familiar with the games in general, the therapist operated the intervention by mixing stages that required different responses to the same stimuli (go/no signal or color) in the go/no-go punch and stepping game; for instance, combining stages 2 and 3, 4 and 5, and 6–9. The therapist combined these stages during a session to stimulate an inhibition response in patients with PD. To increase the level of the challenge, the therapist also had the option of adjusting the number of trials. To increase the difficulty of the number punch game, the therapist increased the number of circles or combined the command types. Participants underwent VR intervention for a total of 10 sessions, 2–3 times a week, for 30 min per session. In every session, all three exergames were performed for 10 min each.

### Outcome measures

The baseline characteristics of the patients, including age, sex, disease duration, H&Y stage, and freezing subtypes, were collected [30]. The feasibility of the intervention was measured by the level of compliance with the study protocol. We also acquired the success rate of the games and accomplished stages by session. The simulator sickness questionnaire (SSQ), developed to measure motion sickness, consists of 16 items and was evaluated before the intervention, after the first session, and after the last (tenth) session [31]. The SSQ is rated on a 4-point ordinal scale; the higher the score, the more negative the result. At the end of the study, participants answered a self-reported questionnaire comprised of 9 items about the intervention (overall satisfaction, improvement in symptoms of PD, interest, motivation, difficulty, comfort, safety, intent to continue training, and expectations in the potential of VR for rehabilitation) and rated them on a 7-point Likert-type scale.

The clinical outcome measures included changes in the Timed Up and Go (TUG) test, percentage of dual-task interference in TUG, Berg balance scale (BBS), UPDRS, Stroop test, trail-making test, and digit span. The participants performed the TUG test under single and two types (cognitive and physical) of dual-task conditions. In the single-task condition, each participant was asked to stand from a chair, walk to a traffic cone (3 m away) at a comfortable pace, walk back to a chair, and sit down. In the cognitive dual-task TUG, participants performed the TUG test with serial subtraction by three, starting from a randomly selected number between 50 and 100. The physical dual-task was performed by carrying a cup filled with water in one hand [32]. The average completion times of the two trials in each condition were used for the analysis. We also recorded videos during TUG evaluations and counted the number of steps. Dual-task interference was calculated to investigate the effect of dual-tasks on TUG time [33].

$${\text{Percentage} \, \text{of} \, \text{dual} \, \text{task} \, \text{interference} } (\%) =\frac{\mathrm{\text{Dual} \, \text{task} \, \text{performance}}-{\text{Single} \, \text{task} \, \text{performance}}}{{\text{Single} \, \text{task} \, \text{performance}}}$$

BBS was employed to assess balance, and UPDRS was used to measure the overall symptoms of PD.

Stroop, trail-making, and digit span tests were used to assess cognitive function. The Stroop test evaluates attention, executive function, processing speed, and cognitive flexibility regarding an individual’s ability to inhibit the habitual response [34]. The Stroop test comprises three parts: word, color, and color-word pages. Each page contains 100 items in five columns of 20 items. Participants were required to read the word or color as quickly and correctly as possible in 45 s on the color-word page. The trail-making test is a neuropsychological measure that assesses psychomotor speed, attention, sequencing, mental flexibility, and visual scanning [35]. For trail A, a participant drew a line as quickly as possible by combining numbers from 1 to 25, placed in random order. The time limit was 360 s, and the test ceased when a participant committed five errors. The Korean version of trail B is to connect a line as quickly as possible, alternating between consecutive numbers and letters in the Korean alphabet. For trail B, the time limit was 300 s, and the test was discontinued when a participant made five errors. The digit span (forward and backward) is an immediate recall test to assess attention. In this study, we used raw scores from the digit span tests.

### Statistical analysis

Data are expressed as mean and standard deviation for continuous variables and median and interquartile range (IQR) for ordinal variables. The Wilcoxon signed-rank test was used to evaluate changes before and after the intervention. Statistical significance was set at *p* < 0.05. All statistical analyses were performed using SPSS version 20.0 for Windows (IBM Corp, Armonk, NY, USA).

## Results

**Fig. 3:**
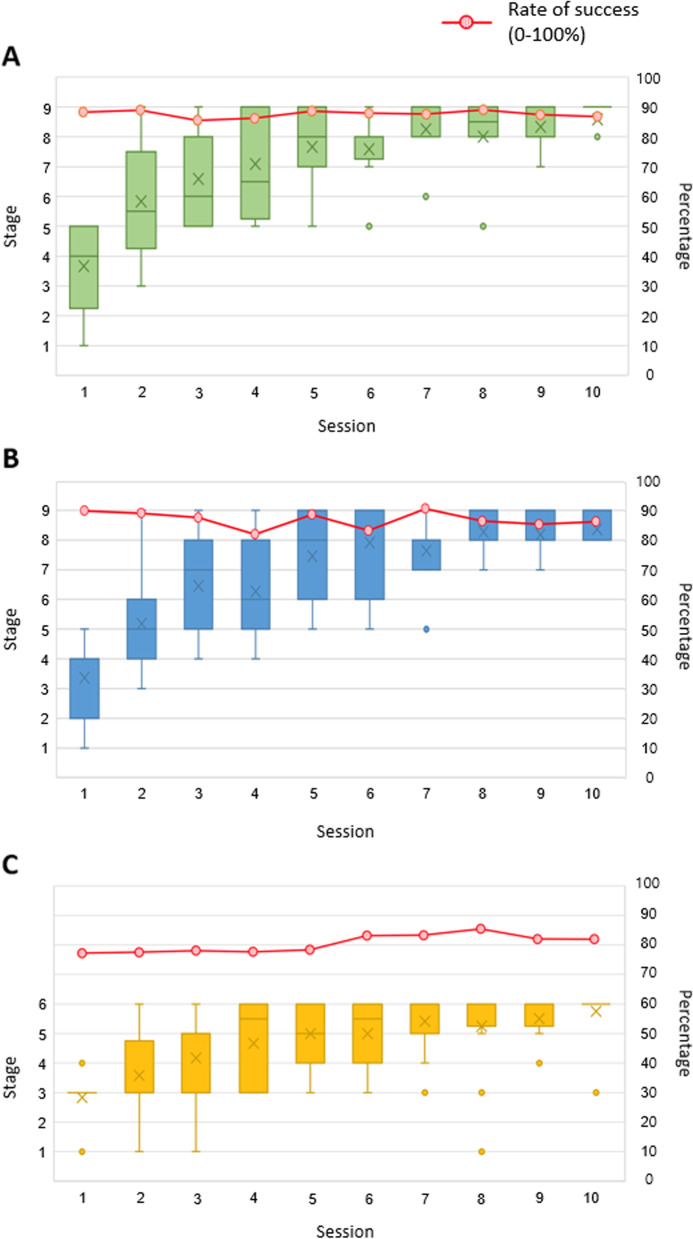
The best performing stage and success rate (%) of each session. **A** The go/no-go punch game. **B** The go/no-go stepping game. **C** The number punch game

In total, 17 patients were screened and 12 patients were enrolled in the study. All participants successfully completed all 10 sessions, each lasting 30 min, without any dropouts. Participants’ characteristics are presented in Table 2. Eleven participants accomplished the highest stage of the games during the intervention period (Fig. 3; Additional file 1: Table S1). The mean success rates of the games per session were 86.90 ± 1.64%, 86.17 ± 2.77%, and 78.96 ± 3.31% in the go/no-go punch, go/no-go stepping, and number punch games, respectively (Fig. 3; Additional file 2: Table S2). The SSQ total scores were 28.05 (IQR: 29.92), 13.09 (IQR: 11.22), and 35.53 (IQR: 52.36) before, after the first session, and after the intervention, respectively (Table 3). Only one adverse event (mild blurred vision), which resolved the following day, was reported. Table 4 shows the results of the participants’ self-reported questionnaire. The overall satisfaction with the intervention was 6.0 (IQR: 1.25) on a 7-point Likert-type scale (Table 4). Participants scored 7.00 (IQR: 0.00) for a sense of safety and 7.00 (IQR: 1.25) for intent to continue training.

**Table 2:** Baseline characteristics of the participants (N = 12)

Male/female (n)		3/9
Age (year)^†^		73.83 ± 6.09
Disease duration (month)^†^		128.83 ± 76.96
Hoehn & Yahr stage 2.5/3 (n)		7/5
Mini-mental state examination-Korea (score)^†^		28.17 ± 1.85
Subtypes in freezing of gait (n)	Non-freezers	5
	Off-freezers	4
	On–off freezers	3

^†^Mean ± SD

**Table 3:** Changes in the scores in the simulator sickness questionnaire

No	Age	Sex	DD (mo)	Before intervention				After 1st session				After intervention			
				N	O	D	Total	N	O	D	Total	N	O	D	Total
1	78	F	116	28.62	22.74	13.92	26.18	38.16	15.16	0	22.44	19.08	22.74	41.76	29.92
2	78	F	71	38.16	7.58	13.92	22.44	19.08	7.58	0	11.22	9.54	7.58	27.84	14.96
3	75	F	62	19.08	37.90	27.84	33.66	0	7.58	27.84	14.96	19.08	45.48	41.76	41.14
4	72	F	57	28.62	83.38	55.68	67.32	0	7.58	27.84	18.70	28.62	68.22	69.60	59.84
5	69	F	183	9.54	15.16	0	11.22	28.62	0	0	11.22	47.70	68.22	69.60	67.32
6	83	M	196	47.70	60.64	55.68	63.58	9.54	45.48	41.76	41.14	9.54	7.58	0	7.48
7	78	F	185	28.62	22.74	27.84	29.92	38.16	7.58	0	11.22	38.16	68.22	41.76	59.84
8	60	M	107	0	0	0	0	0	0	0	0	0	0	0	0
9	78	F	59	0	0	0	0	0	0	0	0	0	0	0	0
10	68	F	102	38.16	60.64	0	44.88	0	0	0	0	9.54	0	13.92	7.48
11	74	F	318	76.32	60.64	41.76	71.06	19.08	37.9	69.6	48.62	47.70	60.64	55.68	59.84
12	73	M	102	19.08	22.74	13.92	22.44	9.54	22.74	13.92	18.70	57.24	60.64	55.68	67.32
Median (IQR)				28.62 (21.47)	22.74 (47.38)	13.92 (31.32)	28.05 (29.92)	9.54 (21.47)	7.58 (17.06)	0 (27.84)	13.09 (11.22)	19.08 (31.01)	34.11 (56.85)	41.76 (45.24)	35.53 (52.36)
*P*				–	–	–	–	0.058	0.011*	0.238	0.008*	0.723	0.933	0.054	0.959

DD, disease duration; IQR, interquartile range; N, nausea; O, oculomotor; D, disorientation

*p < 0.05 by Wilcoxon signed-rank test (compared with pre-intervention score)

**Table 4:** Participants’ self-reported questionnaire results based on a 7-point Likert-type scale (the higher the score, the more positive the result)

Subscales	Score^†^
Overall satisfaction	6.00 (1.25)
Improvement in symptoms of PD	5.50 (2.00)
Interest	6.00 (2.00)
Motivation	6.50 (1.25)
Difficulty	6.00 (2.25)
Comfort	6.00 (3.25)
Safety	7.00 (0.00)
Intent to continue training	7.00 (1.25)
Expectation in use of VR for rehabilitation	6.00 (1.25)

^†^Median (IQR)

Table 5 shows the changes in clinical outcome variables. All outcome measures, except for trail B in the trail-making test, were analyzed in all participants; trail B was analyzed in 11 participants because one participant was unable to finish due to cognitive impairment. Although there was an improvement in the TUG test under single- and dual-task conditions, the differences were not statistically significant. Additionally, the number of steps taken during the TUG test under the physical dual-task condition significantly decreased after the intervention (*p* = 0.045). The BBS score statistically significantly improved from 49.50 (IQR: 5.75) to 51.00 (IQR: 5.00) (*p* = 0.047). A significant improvement was also noted in the color-word test, from 37.50 ± 11.94 to 43.33 ± 10.22 (*p* = 0.003). There was no significant change in the UPDRS, trail-making test, or digit span test.

**Table 5:** Changes in the outcome variables between before and after intervention

		T0	T1	T1–T0
TUG (sec)^†^	Single-task	12.63 ± 3.02	11.95 ± 2.57	0.272
	Dual-task (cognitive)	14.80 ± 2.97	13.82 ± 2.74	0.099
	Dual-task (physical)	20.48 ± 8.06	17.56 ± 4.78	0.136
TUG (the number of steps)^†^	Single-task	19.04 ± 4.26	18.21 ± 3.30	0.332
	Dual-task (cognitive)	21.50 ± 4.85	19.50 ± 3.13	0.109
	Dual-task (physical)	29.08 ± 12.53	24.42 ± 5.15	0.045*
TUG dual-task interference (%)^†^	Cognitive task	18.86 ± 17.70	12.73 ± 13.44	0.136
	Physical task	62.29 ± 51.72	49.30 ± 35.38	0.239
Berg Balance Scale^††^		49.50 (5.75)	51.00 (5.00)	0.047*
UPDRS^††^	Total	38.50 (14.75)	36.50 (11.00)	0.091
	Part I	3.00 (3.00)	2.00 (2.75)	0.058
	Part II	11.50 (3.75)	9.50 (6.75)	0.142
	Part III	20.00 (7.50)	19.50 (8.75)	0.754
	Part IV	4.00 (3.75)	3.50 (3.25)	0.319
Stroop test^†^	Word	76.08 ± 18.61	80.17 ± 20.47	0.059
	Color	63.33 ± 13.07	66.17 ± 15.46	0.146
	Word-color	37.50 ± 11.94	43.33 ± 10.22	0.003*
Trail-making test (sec)^†^	A	46.16 ± 27.27	47.49 ± 25.60	0.347
	B (n = 11)	147.62 ± 101.83	158.84 ± 92.67	0.790
Digit span^†^	Forward	6.58 ± 1.56	6.67 ± 1.72	0.783
	Backward	4.25 ± 1.55	4.08 ± 1.62	0.317

T0, before intervention; T1, after intervention; TUG, timed up and go; UPDRS, unified Parkinson’s disease rating scale

^†^Mean ± SD, ^††^Median (IQR), *p < 0.05 by Wilcoxon signed-rank test

## Discussion

We developed three exergames that combined physical and cognitive tasks in a fully immersive VR environment and investigated their feasibility in patients with PD. High level of compliance with the intervention was observed. In addition, there were few adverse events. These aspects suggest the therapeutic potential of a fully immersive VR application for older patients with PD. Moreover, participants reported high overall satisfaction, sense of safety, and intention to continue training. VR exergames led to improvements in the number of steps taken, balance, and executive function. Although not statistically significant for all outcomes, there were tendencies toward improved motor function.

Many studies have demonstrated the positive effects of VR on motor, cognitive function, and quality of life in PD [23, 36]. Regarding balance, a systematic review showed an average increase of approximately 1.22 points in the BBS immediately after VR therapy in patients with PD, comparable to the 1.5-point improvement in our study [37]. Although the change in score was lower than the clinical detectable change for BBS (2.8–6.6 points) [38], a ceiling effect should be considered because participants in our study had relatively mild static balance impairment at baseline, with a median BBS score of 49.5. Punching gestures in diverse directions that rotate the trunk and combine agile arm motion with posture control might contribute to improving balance [39]. Several studies have shown that VR training improves gait speed, stride length, TUG test results, and UPDRS [40–42]. Likewise, this study showed a decrease in the number of steps under a dual-task condition and trends toward improvement in the TUG test and UPDRS. However, more intensive motor components in the exergames may be considered to ensure statistically significant improvements. Few studies have reported improvements in cognitive function, such as the Montreal Cognitive Assessment, after Nintendo Wii™-based motor and cognitive training [40]. However, limited studies have verified the cognitive effects of VR in patients with PD.

Various types of VR-based rehabilitation have been applied to patients with PD. The most commonly used interventions are Nintendo Wii™ (Nintendo Co., Ltd., Kyoto, Japan) and Xbox^TM^ Kinect (Microsoft Corp., Redmond, WA, USA) [43]. Dual-task training has been performed as a combination of two types of intervention, such as a non-immersive VR maze game (DFKI, Germany) on a balance board (Nintendo Co., Ltd., Kyoto, Japan) [44] or treadmill training with virtual obstacles [45]. However, most studies have used non-immersive or semi-immersive VR hardware and commercial game programs that are not customized for patients with PD. Therefore, we developed a tailored VR exergame including dual-task components based on the pathophysiology of PD.

Dysfunction of the inhibitory response in PD occurs due to the disruption of cortico-basal ganglia circuits that respond to dopamine [46]. Therefore, patients with PD experience difficulties in controlling motor impulsivity, such as initiating movement and stopping ongoing behavior. Exercises with secondary cognitive tasks, such as the go/no-go boxing game, have been utilized to suppress predominant responses and overcome stimulus–response compatibility [47]. In this study, go/no-go exergames require patients to actively use their cognitive effort to act correctly through responding behaviors that were changed according to “go” and “no” or colors at each stage. Repetitive training to inhibit voluntary movements might improve the inhibition response and executive function, which is in line with some studies that confirmed the effects of dual-task training on executive function in older adults, patients with Alzheimer’s disease, and patients with PD [48–51].

The VR exergames in this study may have the advantages of fully immersive VR as well as dual-task training. Fully immersive VR provides patients with a higher “sense of presence,” allowing a more enriched sensorimotor experience [52]. Exergames augmented with multisensory feedback make use of dopaminergic reward systems that can improve brain plasticity or be beneficial to patients with PD [53]. Although cybersickness is a potential safety issue of fully immersive VR, we confirmed that VR is applicable to older patients if adequately designed, similar to the results of a previous study [54]. The initial SSQ score in this study was higher than the absolute value provided by Kennedy et al. [31]. However, it is important to note that the subjects in that study were healthy adults, which makes it inappropriate to directly compare the results to elderly PD patients in this study. Patients with PD encounter various non-motor symptoms, including fatigue, dribbling of saliva, nausea, cognitive dysfunction, sweating, blurred vision, and vertigo [55]. These symptoms partially overlap with the cybersickness symptoms targeted by the SSQ. To ensure that the intervention did not exacerbate the symptoms, we compared the SSQ scores before and after the intervention and observed no significant changes; participants reported high levels of safety, and only one mild adverse event occurred in our study. In addition to the potential adverse effects, it is essential to consider digital literacy, socioeconomic status, and cultural background in order to facilitate the adoption of new technologies among the elderly [56]. The participants demonstrated high levels of interest, motivation, and intent to continue training, thereby confirming their acceptance of the exergames developed in this study.

This study has several limitations. First, it was a feasibility study with a small sample size and no control group. Further large-scale randomized controlled trials are needed to verify the clinical effects of VR exergames in patients with PD. Second, the intensity of exergames (total of 5 h for 3–4 weeks) might be insufficient to improve all clinical outcomes. Generally, 2–3 h of exercise per week for 6–14 weeks is considered an intensive treatment for PD [57]. High-intensity exercise promotes activity-dependent neuroplasticity that results in improvements in motor function, including gait parameters and functional performance [58, 59]. Further studies need to consider higher and longer intervention intensities to confirm the effects of exergames. Third, our VR exergames need to be improved to achieve better therapeutic results. Considering its limited impact on motor function, additional dynamic dual-task training content should be developed. Although only exergames in sitting positions were investigated for patient safety in this study, dynamic training using a harness while standing can be developed in the future. Also, PD is a neurodegenerative condition that requires prolonged and intensive rehabilitation therapies beyond the scope of the protocol discussed. Therefore, it is imperative to consider the development of advanced difficulty levels to improve motor and cognitive functions. Various VR exergames suitable for each PD stage should be developed for clinical application.

## Conclusions

Our feasibility study suggests that fully immersive VR exergames combined with physical and cognitive tasks could be used to train patients with PD without causing serious adverse events. Furthermore, VR exergames may improve executive function and balance. Further development of VR training content is required to achieve better outcomes in motor function and dual-task performance. Large-scale randomized controlled trials are warranted to verify the clinical efficacy of VR exergames for patients with PD.

## Supplementary Information

**Additional file 1: Table S1.** The best performing stage of games by sessions.

**Additional file 2: Table S2.** The success rate of games by sessions.

## Data Availability

The datasets used and/or analyzed during the current study are available from the corresponding author upon reasonable request.

## Abbreviations

BBS: Berg balance scale
HMD: Head-mounted display
H&Y stage: Hoehn and Yahr stage
MMSE: Mini-mental state examination
PD: Parkinson’s disease
SSQ: Simulator sickness questionnaire
TUG: Timed up and go
UPDRS: Unified Parkinson’s disease rating scale
VR: Virtual reality
