# Supplementary material for: Fully immersive virtual reality exergames with dual-task components for patients with Parkinson’s disease: a feasibility study
Source: J Neuroeng Rehabil. 2023 Jul 18;20:92. doi: 10.1186/s12984-023-01215-7 (PMC10355082; doi:10.1186/s12984-023-01215-7)
Supplement: Supplementary file 1 — Additional file 1: Table S1. The best performing stage of games by sessions. [file 12984_2023_1215_MOESM1_ESM.docx]

Table S1. The best performing stage of games by sessions.

|  | Sessions | | | | | | | | | |
| --- | --- | --- | --- | --- | --- | --- | --- | --- | --- | --- |
|  | 1 | 2 | 3 | 4 | 5 | 6 | 7 | 8 | 9 | 10 |
| Go/no-go punch game | 4.00  (2.25) | 5.50  (1.75) | 6.00  (3.00) | 6.50  (3.25) | 8.00  (2.00) | 8.00  (0.25) | 9.00  (1.00) | 8.50  (1.00) | 9.00  (1.00) | 9.00  (0.00) |
| Go/no-go steeping game | 4.00  (1.50) | 5.00  (2.00) | 7.00  (3.00) | 6.00  (3.00) | 8.00  (3.00) | 9.00  (2.50) | 8.00  (0.50) | 9.00  (1.00) | 9.00  (1.00) | 9.00  (1.00) |
| Number punch game | 3.00  (0.00) | 3.00  (1.25) | 5.00  (2.00) | 5.50  (3.00) | 5.00  (2.00) | 5.50  (2.00) | 6.00  (1.00) | 6.00  (0.25) | 6.00  (0.25) | 6.00  (0.00) |

Median (Interquartile range)
