# Supplementary material for: Fully immersive virtual reality exergames with dual-task components for patients with Parkinson’s disease: a feasibility study
Source: J Neuroeng Rehabil. 2023 Jul 18;20:92. doi: 10.1186/s12984-023-01215-7 (PMC10355082; doi:10.1186/s12984-023-01215-7)
Supplement: Supplementary file 2 — Additional file 2: Table S2. The success rate of games by sessions. [file 12984_2023_1215_MOESM2_ESM.docx]

Table S2. The success rate of games by sessions (%)

|  | Sessions | | | | | | | | | | Average |
| --- | --- | --- | --- | --- | --- | --- | --- | --- | --- | --- | --- |
|  | 1 | 2 | 3 | 4 | 5 | 6 | 7 | 8 | 9 | 10 |  |
| Go/no-go punch game | 86.64 ± 10.91 | 89.04 ± 7.09 | 84.68 ± 8.39 | 84.90 ± 11.83 | 87.42 ± 11.14 | 85.06 ± 16.27 | 87.62 ± 11.24 | 89.03 ± 9.13 | 88.22 ± 9.67 | 86.39 ± 11.02 | 86.90 ± 1.64 |
| Go/no-go steeping game | 89.65 ± 6.02 | 88.03 ± 10.79 | 87.59 ± 6.29 | 81.33 ± 12.70 | 86.77 ± 11.34 | 82.35 ± 12.16 | 89.36 ± 6.86 | 86.34 ± 10.09 | 85.72 ± 10.90 | 84.54 ± 11.04 | 86.17 ± 2.77 |
| Number punch game | 76.56 ± 13.29 | 75.05 ± 16.03 | 75.30 ± 15.46 | 78.37 ± 15.69 | 76.08 ± 15.99 | 84.25 ± 13.72 | 81.57 ± 13.67 | 83.41 ± 11.66 | 78.99 ± 15.27 | 79.77 ± 16.68 | 78.96 ± 3.31 |

Mean ± Standard deviation
